# Yes, we have bananas (and also health statistics data): Brazilian-based Medicine

**DOI:** 10.1590/S1516-31802000000500001

**Published:** 2000-09-01

Carmen Miranda was the most important Brazilian singer just before and after World War II. She was also very popular in the United States, singing and dancing wearing a strange hat full of pineapples and bananas. It was one of the many stereotypes about Brazil and Latin American created by Hollywood film-makers.

In the same way, the epidemiological profile of the Brazilian population has been blurred over the decades with the stereotype of being a non-industrialized country (when in fact gross industrial production surpassed agricultural production during the 1930's), and being a place with serious “ tropical diseases”. Curiously, although infectious diseases were until recently considered to be the most important component of mortality in Brazil, cardiovascular diseases have been the main cause of deaths for the whole country since the 1960's (and the primary cause of death in São Paulo and Rio de Janeiro since the 1940's).

In contrast to this stereotype, a seminal paper in Circulation, the leading cardiology journal,^1^ on the emerging epidemic of cardiovascular disease in developing countries, has emphasized the epidemiology of cardiovascular diseases in Brazil from a different viewpoint in comparison with countries in Asia or Africa. The authors cited data obtained from the official health statistics of Brazil that were used in theses^2,3^ and published in Brazilian journals.^4–6^ All of these were summarized in a publication by the InterAmerican Heart Foundation.^7^

In this paper, the authors have stressed a point of view that the profile of cardiovascular disease varies between developing countries, thus: “ the State of São Paulo, in Brazil, experienced declines of 33.6% for men and 40.6% for women in age-standardized cardiovascular diseases rates between 1970 and 1992. Despite this decrease, the mortality rates in the 45-64-year-old age group in São Paulo, Porto Alegre and Rio de Janeiro are reported to be as high as or nearly equal to rates in Eastern Europe. The cumulative mortality rate from coronary heart disease is 42% for Brazilian men below the age of 65 years compared with 25% in industrialized countries. Death from acute myocardial infarction in Brazilian men between the ages of 35 and 44 years is stated to be three times higher than in the United States or Canada”.^1^ This is a good example of how the official health statistics can be used to describe our reality and compare Brazil with other countries without putting into the same basket countries like Argentina and Angola with different socioeconomic development, or like Mexico and India with distinct cultural traditions. Thus, it becomes possible to postulate a new type of epidemiological transition without using stereotypes like “developed countries = declining heart disease mortality, vs. emerging countries = rising cardiovascular disease burden”.

This is all very interesting, as foreign researchers have been able to consider a point of view that does not have acceptance in the Brazilian public health agenda. The Ministry of Health does not have an official policy for the most important killer in this country, despite having spent more than $1 billion on medicines for AIDS.

This fact has occurred because both Brazilian physicians and scientists have had a serious problem in studying our people and our diseases. Their preference for referring to American or European data is a serious cultural problem among well-educated people here. It is a problem “…that merits consideration as the global economy bears down in Brazil and feeds a long-held love of what is foreign”.^8^

Over the recent history of Brazil, one explanation for this habit has been the lack of statistical data about Brazil. This is partially true because during the military dictatorship, some health statistics were considered as a “national security affair”. However, since the mid-1990's, the databank of the Brazilian Ministry of Health has been available on line, and it is now more common to see papers addressing the epidemiological profile of Brazil. However, even today Brazilian scientists are referring to these papers less frequently in comparison with citations of articles addressing epidemiological profiles from overseas.

Although a good improvement in health statistics has been observed, the Brazilian databank presents some limitations due to lack of medical care in rural areas, and surprisingly, a high proportion of ill-defined deaths certified in the city of Rio de Janeiro since the early 1990's due to vagaries in local laws. As the coverage of the mortality system over the whole country is lower than 90 percent and the proportion of ill-defined cases is nearly 20 percent for the whole country (against almost 100 percent coverage and less than 5 percent of ill-defined deaths in the main cities of Brazil), studies that target using these rates must be evaluated cautiously. Most of them do not lend themselves to temporal analyses and international comparisons when the whole country is considered in analyses. On the other hand, other analyses from the states of Sao Paulo and Rio Grande do Sul, and from the main metropolitan areas, are a good surrogate for the comprehension of Brazilian epidemiological profiles. The appropriate use of this data requires some skills in health statistics and the correct definition of the populations and diseases to be analyzed. A good start would be to read a seminal book on health statistics^9^ and other books that have described the epidemiology of chronic diseases in Brazil.^10–12^

To improve the quality of medical communication and to spread the use of Brazilian health statistics data, the São Paulo Medical Journal, Diagnóstico & Tratamento, and the official site of the Associação Paulista de Medicina are launching a series of articles related to our reality. This series will be called Brazilian-based Medicine. When the printed version of this issue of the Journal has been mailed, an article on “Uses and Misuses of Mortality Data” will be made available for reading on our website. In the next issue of the Journal, an important review of Infant Mortality will be published. Our intention is to show to Brazilian physicians and all scientists around the world (the Journal can be reached through the Scielo site: www.scielo.br) how Brazilians live “from the womb to the tomb”.

Finally, as Carmen Miranda sang half a century ago: Yes, we have bananas (and also health statistics data).
